# Supplementary material for: An ACE2 Triple Decoy that neutralizes SARS-CoV-2 shows enhanced affinity for virus variants
Source: Sci Rep. 2021 Jun 17;11:12740. doi: 10.1038/s41598-021-91809-9 (PMC8211782; doi:10.1038/s41598-021-91809-9)
Supplement: Supplementary file 1 — Supplementary Information. [file 41598_2021_91809_MOESM1_ESM.pdf]

SUPPLEMENTARY INFORMATION FOR

An ACE2 Triple Decoy that neutralizes SARS-CoV-2 shows enhanced affinity for virus variants

Shiho Tanaka, Gard Nelson, Anders Olson, Oleksandr Buzko, Wendy Higashide, Annie Shin, Marcos Gonzales, Justin Taft, Roosheel Patel, Sofija Buta, Ashley Richardson, Dusan Bogunovic, Patricia Spilman, Kayvan Niazi, Shahrooz Rabizadeh, and Patrick Soon-Shiong

Results

Table S1. ***BLI kinetics values for ACE2(WT) decoys***

Ligand	Analyte	kon1 (1/Ms)	kon2 (1/Ms)	koff 1 (1/s)	koff 2 (1/s)	KD (nM)
ACE2-IgG1Fc	RBD	3.46E+05		7.41E-03		21.4
RBD	ACE2-IgG1Fc	1.03E+05	1.89E+00	7.89E-05	3.02E-01	0.762
RBD	ACE2-IgAFc	5.17E+04	3.90E+00	8.59E-06	1.06E+00	0.166

Table S2 ***BLI kinetics values and*** IC_{50} ***in the live virus assay***

Decoy	kon (1/Ms)	koff (1/s)	KD (nM)	Virus neutralization IC_{50} (µg/mL)
ACE2(WT)-IgG1Fc	3.46E+05	7.41E-03	21.4	1.614
ACE2(T27Y)-IgG1Fc	5.05E+05	4.05E-03	8.01	N/D
ACE2(H34A)-IgG1Fc	2.85E+05	1.17E-03	4.09	N/D
ACE2(T27Y/H34A)-IgG1Fc	3.77E+05	2.11E-04	0.56	0.107

As shown in Figure S1, wild type (WT) and the T27Y/H34A mutations had similar ACE2 enzymatic activity. Addition of R273Q, R273K, R273L, H245A, H505L, H374N, or H378N mutations in combination with the T27Y/H34A mutations inhibited activity of ACE2.

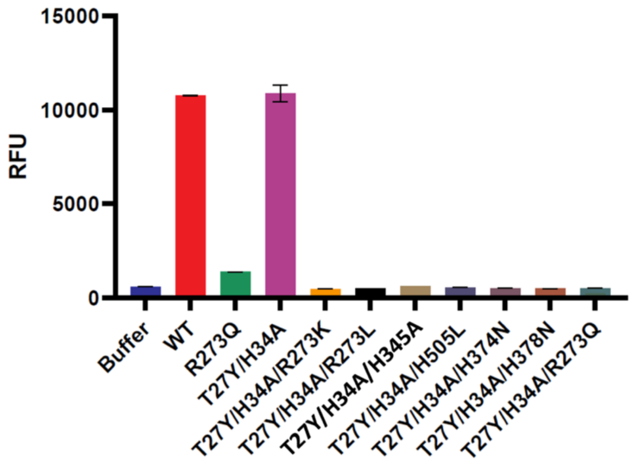

Fig. S1. *ACE2 activity assay*. Enzyme activity of ACE2 in relative fluorescent units (RFU) for each decoy is shown.

To choose which of these activity-inhibiting mutations would be used in combination with the two affinity-enhancing mutations, we compared BLI kinetic analysis of S RBD binding for each. Of the triple mutants, the ones expressing R273K or H374N had the lowest dissociation coefficient (K_D), that is, highest affinity binding.

The T27Y/H34A/H374N triple mutant was chosen for further testing because it showed better biophysical properties, including a lower propensity to aggregate, higher titer/better Tm as compared to the decoy with the R273K substitution.

Table S3. ***BLI analysis of S RBD binding by triple mutants.***

ACE2 Mutants	kon (1/Ms)	koff (1/s)	KD (nM)
T27Y/H34A	3.77E+05	2.11E-04	0.56
T27Y/H34A/R273K	4.04E+05	1.79E-04	0.44
T27Y/H34A/R273L	4.06E+05	4.04E-04	1.00
T27Y/H34A/H345A	4.15E+05	3.95E-04	0.95
T27Y/H34A/H505L	4.09E+05	2.91E-04	0.71
T27Y/H34A/H374N	3.88E+05	2.44E-04	0.63
T27Y/H34A/H378N	3.95E+05	3.32E-04	0.84
T27Y/H34A/R273Q	4.86E+05	4.09E-04	0.84

Titer analysis for the triple decoys is shown below in Fig. S2 and Table S4.

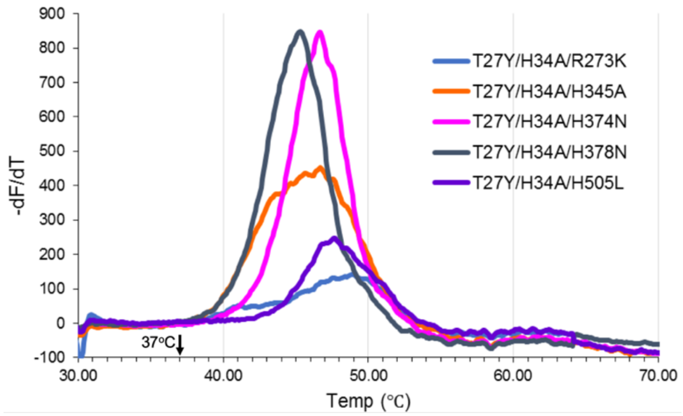

Fig. S2. T_m analysis.

Table S4. ***Tm analysis.***

ACE2 mutants	Day 7 Titer (µg/mL)	% main peak post-ProA purification
T27Y/H34A/R273K	10.6	55.7
T27Y/H34A/H345A	27.3	70.8
T27Y/H34A/H374N	23.2	82.7
T27Y/H34A/H378N	30.1	75.8
T27Y/H34A/H505A	5.3	70

The BLI kinetics analysis and binding values for ACE2 WT binding to naturally-occurring B.1.351 and CAL.20C variants are shown in Fig. S3 and Table S5.

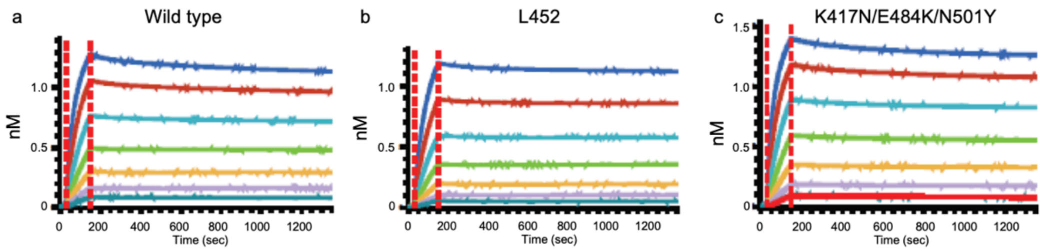

Fig. S3 *BLI kinetic analysis of ACE wild type (WT) decoy against SARS-CoV-2 RBD WT and L452R and K417N/E484K/N501Y mutants with avidity.*

Table S5. ***BLI kinetic analysis of ACE2(WT) and ACE2 Triple Decoy binding to S RBD WT and variants***

	**ACE2(WT)-IgG1Fc**			**ACE2(T27Y/H34A/H374N)-IgG1Fc**		
RBD mutants	kon (1/Ms)	koff (1/s)	KD (nM)	kon (1/Ms)	koff (1/s)	KD (nM)
Wild-type	5.25E+05	4.90E-03	9.33	6.91E+05	2.17E-04	0.315
E484K	8.00E+05	7.02E-03	8.77	1.00E+06	3.95E-04	0.393
K417N	3.97E+05	1.35E-02	34	4.46E+05	9.33E-04	2.09
K417N/E484K	4.84E+05	1.73E-02	35.7	8.03E+05	1.10E-03	1.37
N501Y	6.38E+05	1.45E-03	2.27	9.50E+05	1.40E-04	0.148
E484K/N501Y	8.26E+05	1.76E-03	2.13	1.22E+06	1.16E-04	0.095
K417N/N501Y	4.57E+05	3.47E-03	7.6	7.47E+05	3.34E-04	0.447
K417N/E484K/N501Y	7.45E+05	3.93E-03	5.28	7.56E+05	3.51E-04	0.465
L452R	4.39E+05	1.19E-03	2.71	4.62E+05	1.02E-04	0.221

Table S6. ***BLI kinetic analysis of ACE(WT) decoy:S RBD mutant binding with avidity.***

**RBD Ligand**	**Analyte**	**kon1 (1/Ms)**	**kon2 (1/Ms)**	**koff 1 (1/s)**	**koff 2 (1/s)**	**KD (nM)**
Wild type (WT)	ACE2-IgG1Fc	1.25E+05	2.70E+00	1.38E-04	3.30E-01	1.11
L452R	ACE2-IgG1Fc	8.50E+04	2.71E+00	5.03E-05	5.92E-01	0.592
K417N/E484E/N501Y	ACE2-IgG1Fc	1.91E+05	2.56E+00	8.67E-05	9.62E+01	0.453

Table S7. ***Binding of 'theoretical' S RBD variant to the ACE2(WT) decoy***

RBD Mutants	ACE2 WT Decoy KD (nM)
Wild-type	9.33
K417N	34.00
E484K	8.77
N501Y	2.27
E484K/N501Y	2.13
K417N/E484K	35.70
K417N/N501Y	7.60
K417N/E484K/N501Y	5.28
L452R	5.58
L452R/K417N	15.20
L452R/E484K	4.10
L452R/N501Y	1.22
L452R/K417N/E484K/N501Y	4.00

The time series showing full sampling of both CVs from the ACE2 WT:RBD WT simulation is shown in Figure S4.

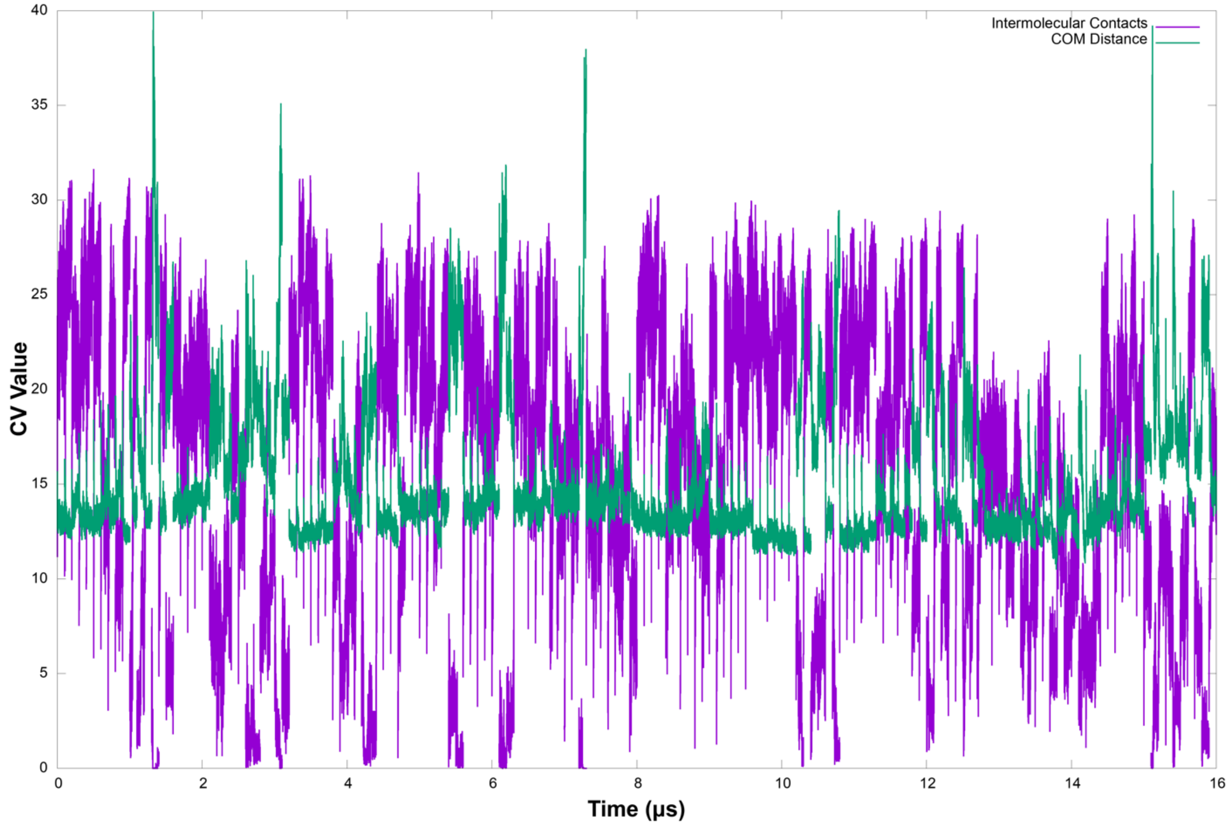

Fig. S4 *Time series of both collective variables (CV) used in the adaptively biased molecular dynamics (ABMD) simulation*. ABMD walkers were started at multiple CV values and allowed to sample conformation space. The full range of both CVs was sampled and multiple transitions across the bound/unbound dividing line were observed.
